# Coordinated Expression of Phosphoinositide Metabolic Genes during Development and Aging of Human Dorsolateral Prefrontal Cortex

**DOI:** 10.1371/journal.pone.0132675

**Published:** 2015-07-13

**Authors:** Stanley I. Rapoport, Christopher T. Primiani, Chuck T. Chen, Kwangmi Ahn, Veronica H. Ryan

**Affiliations:** 1 Brain Physiology and Metabolism Section, Laboratory of Neurosciences, National Institute on Aging, National Institutes of Health, Bethesda, MD, United States of America; 2 Section on Nutritional Neurosciences, National Institute on Alcohol Abuse and Alcoholism, National Institutes of Health, Bethesda, MD, United States of America; 3 Child Psychiatry Branch, National Institute of Mental Health, National Institutes of Health, Bethesda, MD, United States of America; Florida International University, UNITED STATES

## Abstract

**Background:**

Phosphoinositides, lipid-signaling molecules, participate in diverse brain processes within a wide metabolic cascade.

**Hypothesis:**

Gene transcriptional networks coordinately regulate the phosphoinositide cascade during human brain Development and Aging.

**Methods:**

We used the public BrainCloud database for human dorsolateral prefrontal cortex to examine age-related expression levels of 49 phosphoinositide metabolic genes during Development (0 to 20+ years) and Aging (21+ years).

**Results:**

We identified three groups of partially overlapping genes in each of the two intervals, with similar intergroup correlations despite marked phenotypic differences between Aging and Development. In each interval, *ITPKB*, *PLCD1*, *PIK3R3*, *ISYNA1*, *IMPA2*, *INPPL1*, *PI4KB*, and *AKT1* are in Group 1, *PIK3CB*, *PTEN*, *PIK3CA*, and *IMPA1* in Group 2, and *SACM1L*, *PI3KR4*, *INPP5A*, *SYNJ1*, and *PLCB1* in Group 3. Ten of the genes change expression nonlinearly during Development, suggesting involvement in rapidly changing neuronal, glial and myelination events. Correlated transcription for some gene pairs likely is facilitated by colocalization on the same chromosome band.

**Conclusions:**

Stable coordinated gene transcriptional networks regulate brain phosphoinositide metabolic pathways during human Development and Aging.

## Introduction

Phosphoinositides, inositol-containing derivatives of phosphatidic acid that lack nitrogen, participate in neurotransmission, autophagy, apoptosis, neuronal and glial growth, myelination, and membrane trafficking in brain [1–3]. Their participation is highly energy dependent [1–3]. Phosphoinositide metabolism is disturbed in many human brain diseases [1, 4–7] and in animal models for some of these diseases [8–10]. Changes in phosphoinositide metabolites and enzymes also accompany normal human brain development and aging [4–7, 11, 12].

The complexity of brain phosphoinositide metabolism limits our understanding the roles of phosphoinositides in Development and Aging and our ability to design therapeutic interventions in disease states [10, 13–17]. One way to address these limitations may be to analyze age-related transcription of phosphoinositide genes in brain over the lifespan. During Development (0 to ~20 years), the human brain undergoes marked nonlinear changes in synaptic and dendritic growth and pruning, neuronal loss, glial elaboration and myelination, in arachidonic and docosahexaenoic acid concentrations, and it shifts from ketone body to glucose consumption for ATP synthesis [18–24]. During later Aging (21+ years), brain function and metabolism are maintained in a more homeostatic range, although risk for neurodegeneration increases [25].

Several databases are available to examine age changes in gene expression in the human brain, including the publically accessible BrainCloud for the dorsolateral prefrontal cortex (http://braincloud.jhmi.edu) [26–28]. We recently used BrainCloud to demonstrate age-related coordinated expression patterns during Development and Aging of genes of phospholipase A_2_ (PLA_2_)-initiated arachidonic acid (AA, 20:4n-6) and docosahexaenoic acid (22:6n-3) metabolic cascades [29] and of genes for cytokines, chemokines, and other inflammatory proteins [30].

In the present study, we used BrainCloud to compare age-related expression in human dorsolateral prefrontal cortex of 49 genes involved in phosphoinositide synthesis, degradation, and signaling [1, 2]. Based on our prior studies [29, 30], we hypothesized that we could identify coordinated expression of these genes during the Development and Aging intervals. Such changes might correspond to changes in biochemical reactions involving the gene products and be facilitated by colocalization on a chromosomal band [29–34].

## Methods

We selected 49 genes involved in phosphoinositide metabolism, based on canonical pathways reported in Ingenuity Pathway Analysis (IPA) (Ingenuity Systems, Redwood City, CA, http://www.ingenuity.com) and other sources [1, 2]. Expression data for each gene were exported from the BrainCloud database from 231 males and females ranging in age from birth to 78 years [26]. No subject had a history of significant psychiatric, neurological disorder, or drug abuse, or postmortem evidence of neuropathology.

As described in our prior studies, we separated the samples into Development (0 to 20.95 years, 87 subjects) and Aging (21 to 78.2 years, 144 subjects) intervals [29, 30]. Gender and race breakdowns, as well as a description of the data in BrainCloud, have been reported earlier [29, 30].

Twenty-two of the 49 genes chosen were detected by more than one probe in the BrainCloud database. When possible (18 of these 22 genes), the probe covering all possible alternate transcripts of the gene was chosen using the Gene View tab on BrainCloud. The probe that covered all transcripts also was the highest intensity probe for all but one gene (*PIP5K1A*), for which we used the probe covering all the transcripts. If one probe did not cover all possible transcripts, we chose the highest intensity probe (*SLC5A3*, *PTGS2*, and *ITPK1*).

Statistical tests were performed using Partek Genomics Suite (Version 6.6, Partek, St. Louis, MO, USA) and GraphPad Prism 5 (Version 5.02, GraphPad Software, La Jolla, CA, USA). First, a t-test was performed in Partek to determine whether mean expression levels differed significantly between the Aging and Development intervals for each gene. Pearson’s r correlations were performed in GraphPad Prism 5 (Graph Pad Software, La Jolla, CA) for each gene, to determine correlation with age in each interval. Visual observation during Development suggested nonlinear expression for some genes. We therefore compared goodness of fit of the data with a nonlinear equation, Y = (Y0 - Plateau)*exp(-K*A) + Plateau (where Y = expression level at age A, and Y0 expression level at A = 0 years), to goodness of fit with a linear regression between 0 and 20 years [29, 30].

Data also were analyzed with Cluster 3.0 software [35], without filtering or adjustment. As described earlier [29, 30], we calculated distance between probes using the Euclidean distance calculation and clustering using the centroid linkage method expression [36]. The output.cdt file was loaded into TreeView program [37] to generate figures showing relatedness among genes of interest.

Similarity matrices using Pearson’s r correlations and hierarchical clustering also were created using Partek, to make correlation (heat) maps showing correlation coefficients and gene clusters in expression between pairs of genes during Development and Aging [29, 30]. Correlation data from the heat maps were used to construct corresponding statistical significance matrices. GraphPad Prism was used to calculate Pearson’s r correlations for pairs of genes closely located on the same chromosome band.

Ethics Statement. This research was supported entirely by the Intramural Programs of the National Institute on Aging, the National Institute of Alcohol Abuse and Alcoholism, and the National Institute of Mental Health, National Institutes of Health. No author has a conflict of interest. Samples were collected under NIH protocol number NCT00001260, 900142, which include written informed consent from next-of-kin including consent for clinical records to be used. Every brain is consented.

## Results

### Construction of phosphoinositide metabolic pathways

**Fig 1:**
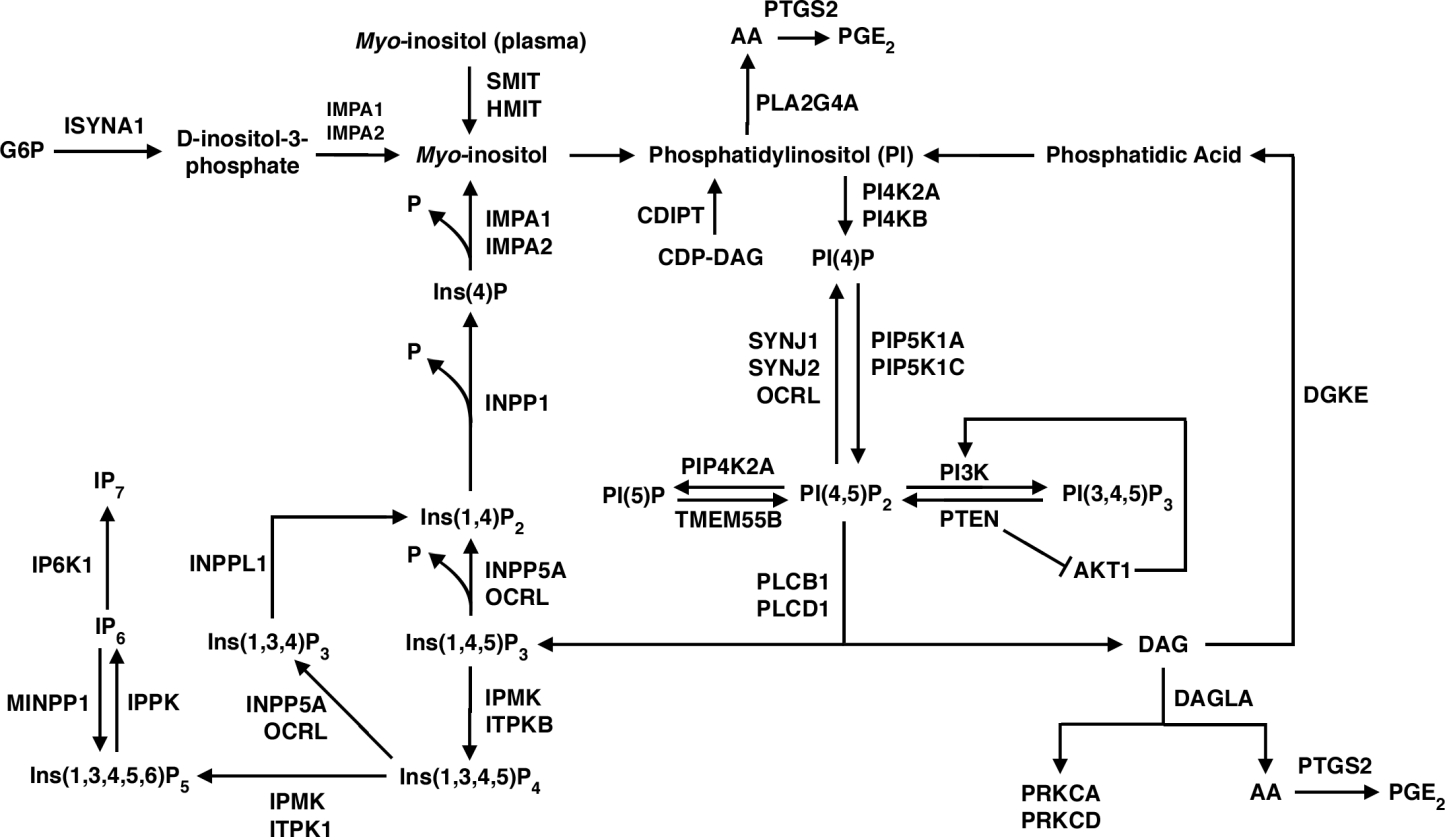
Pathways of phosphoinositide metabolism. See Results section for details.

We constructed pathways of phosphoinositide metabolism within a large cascade, as illustrated in Fig 1, when using ingenuity analysis (http://www.ingenuity.com) and other literature references [1, 2]. In the upper left of Fig 1, inositol-3-phosphate synthase (ISYNA1) is shown to catalyze synthesis of D-inositol-3-phosphate from glucose-6-phosphate (G6P). Then, inositol monophosphatase (IMPA1, IMPA2) hydrolyzes D-inositol-3-phosphate to form *myo*-inositol, which also can be transported into brain from blood by the sodium/*myo*-inositol transporter (SMIT, *SLC5A3*) or the H^+^/*myo*-inositol symporter (HMIT, *SLC2A13*) [1, 38]. CDP (cytidine 5'-diphospho)-diacylglycerol-inositol-3-phosphatidyltransferase (CDIPT) phosphorylates *myo*-inositol to phosphatidyl-1D-*myo*-inositol (phosphatidylinositol, PI) using CDP-diacylglycerol. PI constitutes the largest phosphoinositide pool in brain [2].

Phosphatidylinositol is phosphorylated by PI 4-kinase (PI4KB, PI4K2A) to PI(4)P, which in turn is phosphorylated to PI(4,5)P_2_ by PI(4)P 5-kinase (PIP5K1A, PIP5K1C) [2]. These reactions occur primarily in the endoplasmic reticulum, from where phosphoinositides can be brought to the plasma membrane *via* membrane trafficking, but some reactions also take place at the plasma membrane itself [2]. Reconversion (dephosphorylation) of PI(4,5)P_2_ to PI(4)P can be catalyzed by synaptojanin (SYNJ1, SYNJ2) and Oculocerebrorenal Syndrome Of Lowe (OCRL) [2, 39].

Phospholipase C (PLCB1, PLCD1) is a critical enzyme that integrates phosphoinositide metabolism with neurotransmission, hormonal, and other signaling processes. PLC can be activated by G-protein mechanisms coupled to muscarinic cholinergic M_1,3,5_, dopaminergic D_2,3,4_, serotonergic 5-HT_2A,C_, and other post-synaptic neuroreceptors [40], or to hormone receptors [2]. PLC cleaves membrane PI(4,5)P_2_ to form two important second messengers, cytosol-soluble inositol 1,4,5-trisphosphate (Ins(1,4,5)P_3_, or IP_3_) and membrane-bound diacylglycerol (DAG) [1, 14].

Newly formed IP_3_ moves through the cytosol and binds to IP_3_ receptors on the endoplasmic reticulum, thereby stimulating calcium release [41, 42]. Released calcium creates a positive feedback loop to PLC and to store-operated plasma membrane calcium channels [41, 42]. IP_3_ also can be dephosphorylated by Type I inositol 1,4,5-trisphosphate 5-phosphatase (INPP5A) to Ins(1,4)P_2_. Ins(1,4)P_2_ in turn can be dephosphorylated to Ins(4)P and thence by IMPA1 or IMPA2 to *myo*-inositol. Released *myo*-inositol becomes available for synthesis of PI and the resynthesis of PI (4,5)P_2_ to complete the “PI cycle” (Fig 1) [1, 2, 43].

Additionally, IP_3_ can be converted to highly phosphorylated inositol pyrophosphates like IP_7_, which participate in cell growth and apoptosis [44] (Fig 1, lower left). IP_4_ and IP_5_ are formed primarily by inositol polyphosphate mutikinase (IPMK); deleting *IPMK* in mice prevents formation of IP_5_, IP_6_, and IP_7_ [44, 45].

IPMK also can act as a PI3 kinase (PI3K), which has implications in the mTOR (mammalian target of rapamycin)/protein kinase B (AKT) signaling pathway that participates in growth processes [44]. Inhibition of this pathway, either directly by rapamycin or indirectly by caloric restriction, has been implicated in increased longevity in animal models [46]. Also, apoptosis and autophagy are mediated by phosphatidylinositol 4,5-bisphosphate 3-kinase (PIK3CB, PIK3CA) and AKT [47]. AKT interacts with phosphatidylinositol 3,4,5-trisphosphate 3-phosphatase (PTEN) (Fig 1, center).

The other second messenger released by PLC mediated hydrolysis is membrane-bound DAG, which binds to and activates protein kinase C (PKC) and other kinases that phosphorylate intracellular proteins [2]. DAG can be lost by phosphorylation by diacylglycerol kinase (DGKE) and recycled into phosphatidylinositol (PI), effectively terminating activation of PKC, or it can be hydrolyzed by diacylglycerol lipase (DAGLA) to arachidonic acid (AA). Arachidonic acid in turn can be oxidized by COX-2 (*PTGS2*) to produce pro-inflammatory metabolites like prostaglandin E_2_ (PGE_2_) (Fig 1, lower right) [29, 48]. Arachidonic acid also may be released from PI by calcium-dependent cPLA_2_ type IVA (*PLA2G4A*), then converted to PGE_2_ by COX-2 (Fig 1, top) [29, 31].

### Age correlations in expression of genes and gene pairs

Table 1 lists the 49 genes in this analysis, their corresponding protein names, chromosomal locations, probes used, and results of t-tests comparing mean expression levels between Aging and Development. All fold changes are less than |2|, suggesting relatively stable expression throughout the lifespan. *SYNJ2* has the highest fold increase (1.81, p = 10^−15^) and *SLC2A13* has the largest fold decrease (-1.67, p = 10^−9^) in Aging compared with Development. Correcting for 49 comparisons, at p < 0.001, 14 genes are higher and of 6 genes are lower in mean expression during Aging than Development.

**Table 1:** Gene list with protein name, chromosomal location, and ANOVA comparing mean expression levels between Aging and Development.

Gene Name	Protein Name	Chromosome	Probe	Fold change	p-value
*CYTH3*	General receptor for phosphoinositides	7p22.1	1	-1.16	0.0001
*IMPA1*	Inositol monophosphatase 1	8q21.1-q21.3	1	1.21	3.27E-07
*INPP5A*	Type I inositol 1,4,5-trisphosphate 5-phosphatase	10q26.3	1	1.23	9.44E-07
*IP6K1*	Inositol hexakisphosphate kinase 1	3p21.31	1	1.2	5.19E-07
*ITPKB*	Inositol-trisphosphate 3-kinase B	1q42.12	2	1.62	2.16E-13
*MTM1*	Myotubularin	Xq27.3-q28	2	1.26	1.21E-06
*MTMR14*	Myotubularin-related protein 14	3p26	2	1.14	1.31E-08
*PIK3R4*	Phosphoinositide 3-kinase regulatory subunit 4	3q22.1	1	1.27	8.08E-10
*PIP5K1C*	Phosphatidylinositol 4-phosphate 5-kinase type-1 gamma	19p13.3	2	-1.22	4.64E-07
*PRKCD*	Protein kinase C, delta	3p21.31	1	-1.27	2.99E-06
*SACM1L*	Phosphatidylinositide phosphatase SAC1	3p21.3	1	1.27	1.16E-11
*SLC2A13*	Solute carrier family 2 (facilitated glucose transporter), member 13 (HMIT)	12q12	1	-1.67	1.29E-09
*SYNJ1*	Synaptojanin-1	21q22.2	2	1.32	9.25E-06
*SYNJ2*	Synaptojanin-2	6q25.3	1	1.81	1.85E-15
*TMEM55B*	Type 1 phosphatidylinositol 4,5-bisphosphate 4-phosphatase	14q11.1	1	1.2	9.55E-13
*PIP4K2A*	Phosphatidylinositol 5-phosphate 4-kinase type-2 alpha	10p12.2	2	1.27	0.0005
*PTEN*	Phosphatidylinositol 3,4,5-trisphosphate 3-phosphatase and dual-specificity protein phosphatase	10q23	1	-1.27	0.0004
*PIK3R2*	Phosphatidylinositol 3-kinase regulatory subunit beta	19p13.11	1	-1.12	0.0005
*PRKCA*	Protein kinase C, alpha	17q22-q24	2	1.14	0.0006
*PIK3R1*	Phosphatidylinositol 3-kinase regulatory subunit alpha	5q13.1	2	1.16	0.00071
*ISYNA1*	Inositol-3-phosphate synthase 1	19p13.11	1		ns
*MINPP1*	Multiple inositol polyphosphate phosphatase 1	10q23	2		ns
*PTGS2*	Prostaglandin-endoperoxide synthase 2	1q25.2-q25.3	4		ns
*CDIPT*	CDP-diacylglycerol—inositol 3-phosphatidyltransferase	16p11.2	2		ns
*GRASP*	General receptor for phosphoinositides associated scaffold protein	12q13.13	1		ns
*PI4KB*	Phosphatidylinositol 4-kinase beta	1q21	2		ns
*INPP1*	Inositol polyphosphate 1-phosphatase	2q32	1		ns
*PIK3C3*	Phosphatidylinositol 3-kinase catalytic subunit type 3	18q12.3	1		ns
*IPMK*	Inositol polyphosphate multikinase	10q21.1	1		ns
*INPP4A*	Type I inositol 3,4-bisphosphate 4-phosphatase	2q11.2	2		ns
*PIK3C2B*	Phosphatidylinositol 4-phosphate 3-kinase C2 domain-containing subunit beta	1q32	1		ns
*DAGLA*	Diacylglycerol lipase, alpha	11q12.3	1		ns
*AKT1*	Protein kinase B	14q32.32-q32.33	2		ns
*PI4K2A*	Phosphatidylinositol 4-kinase type 2-alpha	10q24	1		ns
*PIK3R3*	Phosphatidylinositol 3-kinase regulatory subunit gamma	1p34.1	2		ns
*OCRL*	Inositol polyphosphate 5-phosphatase OCRL-1	Xq25	2		ns
*INPPL1*	Phosphatidylinositol 3,4,5-trisphosphate 5-phosphatase 2	11q23	2		ns
*PIK3CB*	Phosphatidylinositol 4,5-bisphosphate 3-kinase catalytic subunit beta isoform	3q22.3	1		ns
*PIP5K1A*	Phosphatidylinositol 4-phosphate 5-kinase type-1 alpha	1q21.3	3		ns
SLC5A3	Solute carrier family 5 (sodium/myo-inositol cotransporter), member 3 (SMIT)	21q22.11	4		ns
PLA2G4A	Phospholipase A2, group IVA (cytosolic, calcium-dependent)	1q25	1		ns
PIK3CA	Phosphatidylinositol 4,5-bisphosphate 3-kinase catalytic subunit alpha isoform	3q26.3	1		ns
PLCB1	1-phosphatidylinositol 4,5-bisphosphate phosphodiesterase beta-1	20p12	2		ns
DGKE	diacylglycerol kinase, epsilon	17q22	2		ns
IPPK	Inositol-pentakisphosphate 2-kinase	9q22.31	1		ns
PLCD1	1-Phosphatidylinositol 4,5-bisphosphate phosphodiesterase delta-1	3p22-p21.3	1		ns
ITPK1	Inositol-tetrakisphosphate 1-kinase	14q32.12	4		ns
IMPA2	Inositol monophosphatase 2	18p11.2	1		ns
PIK3C2A	Phosphatidylinositol 4-phosphate 3-kinase C2 domain-containing subunit alpha	11p15.5-p14	1		ns

The probe column indicates type of probe used: 1, only one probe in database; 2, highest intensity probe covering all transcripts; 3, probe used covers all transcripts but is not highest intensity probe; 4, highest intensity probe as none of the probes covered all transcripts. The ANOVA column shows whether levels in Aging are higher or lower than in Development, after correcting for 49 comparisons (p < 0.001). ns, not significant.

Table 2 shows statistically significant correlations between gene expression levels and age in the two intervals. Correcting for 98 comparisons, at p < 0.0005 fewer age correlations are evident during Aging than Development. Only expression of *ITPKB* (r = 0.34) and of *GRASP* (r = - 0.38) correlates significantly with age during the Aging interval. During Development, on the other hand, expression of *PIP42K2A*, *SYNJ2*, *SACM1L*, *IMPA1*, *PLA2G4A*, *SYNJ1*, *PIK3CB*, *PIK3R4*, *INPP1*, *PRKCD*, *PIK3CD*, *PIK3C3*, and *ITPKB* increase significantly, while expression of *AKT1*, *PIK3R2*, *IPMK*, *CYTH3*, *PIP5K1C*, and *PIK3C2B* decrease significantly.

**Table 2:** Statistically significant correlations of gene expression with age during Development and Aging intervals.

Gene name	Development		Aging	
	Pearson r	p value	Pearson r	p value
*PIP4K2A*	0.66	< 0.0001		
*AKT1*	-0.59	< 0.0001		
*SYNJ2*	0.81	< 0.0001		
*SACM1L*	0.60	< 0.0001		
*IMPA1*	0.60	< 0.0001		
*PLA2G4A*	0.55	< 0.0001		
*SYNJ1*	0.52	< 0.0001		
*PIK3CB*	0.53	< 0.0001		
*PIK3R2*	-0.58	< 0.0001		
*PIK3R4*	0.46	< 0.0001		
*INPP1*	0.45	< 0.0001		
*IPMK*	-0.43	< 0.0001		
*CYTH3*	-0.45	< 0.0001		
*PIP5K1C*	-0.46	< 0.0001		
*PIK3C2B*	-0.40	0.0001		
*PRKCD*	0.39	0.0002		
*PIK3C3*	0.37	0.0004		
*ITPKB*	0.37	0.0004	0.34	< 0.0001
*GRASP*			-0.38	< 0.0001

Statistical significance, corrected for 98 comparisons, taken as p < 0.0005. Nonsignificant values not shown.

**Fig 2:**
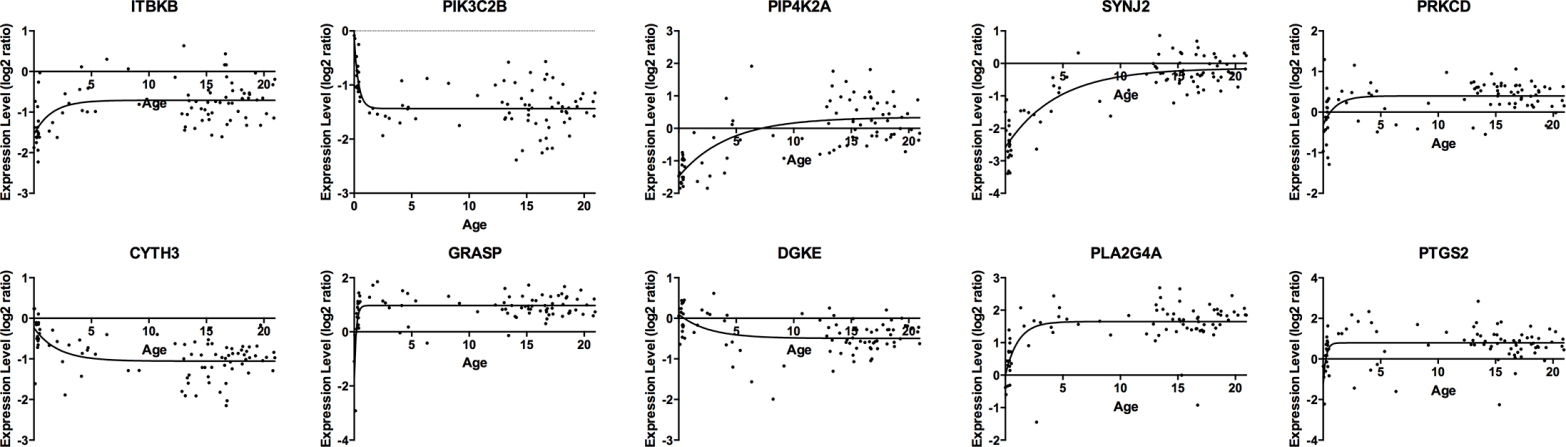
Nonlinear gene expression changes in relation to age during Development, for 10 genes. Data were fit by following equation, Y = (Y0 - Plateau)*exp(-K*A) + Plateau (where Y = expression level at age A, and Y0 expression level at A = 0 years).

Visual observation of expression levels during Development suggested nonlinear changes for some genes. We tested this by comparing nonlinear to linear goodness of fits for each of the 49 genes. For 10 of them, as illustrated in Fig 2, expression of *ITBKB*, *PIP4K2A*, *SYNJ2*, *PRKCD*, *GRASP*, *PLA2G4A* and *PTGS2* increase non-linearly in the first years of life before reaching a plateau, whereas expression of *PIK3C2B*, *CYTH3*, and *DGKE* decline before reaching a plateau.

Colocalization on a chromosome may facilitate transcription of genes whose protein products participate in tightly connected metabolic pathways [29, 32]. To consider this mechanism for our genes, we list in Table 3 statistically significant (p < 0.0001) correlations between genes on the same chromosomal band. On 1q25, expression of *PLA2G4A* (cPLA_2_ Type IVA) correlates with expression of *PTGS2* (COX-2) during both Development and Aging. Genes located on 3p21-23, *PLCD1*, *SACMIL*, *IP6K1*, and *PRKCD*, are significantly correlated with each other during Development and/or Aging. Significant correlations during both intervals occur between *PIK3R4* and *PIK3CB* on 3q22 and between *ITPK1* and *AKT1* on 14q32. During Development *DGKE and PRKCA* are significantly correlated on 17q22.

**Table 3:** Significant correlations between pairs of genes located on the same chromosomal band during the Development and Aging intervals.

			Development		Aging	
Chromosomal Location	Gene	Gene	Pearson's r	p-value	Pearson's r	p-value
1q25	*PLA2G4A*	*PTGS2*	0.58	< 0.0001	0.54	< 0.0001
3p21-23	*SACM1L*	*PLCD1*	-0.41	< 0.0001		
3p21-23	*IP6K1*	*PLCD1*	-0.43	< 0.0001	-0.49	< 0.0001
3p21-23	*PRKCD*	*PLCD1*			-0.52	< 0.0001
3q22	*PIK3R4*	*PIK3CB*	0.48	< 0.0001	0.38	< 0.0001
14q32	*ITPK1*	*AKT1*	0.46	< 0.0001	0.48	< 0.0001
17q22	*DGKE*	*PRKCA*	0.49	< 0.0001		

Nonsignificant correlations not shown.

### Cooperative clustered transcription correlations within extended groups

**Fig 3:**
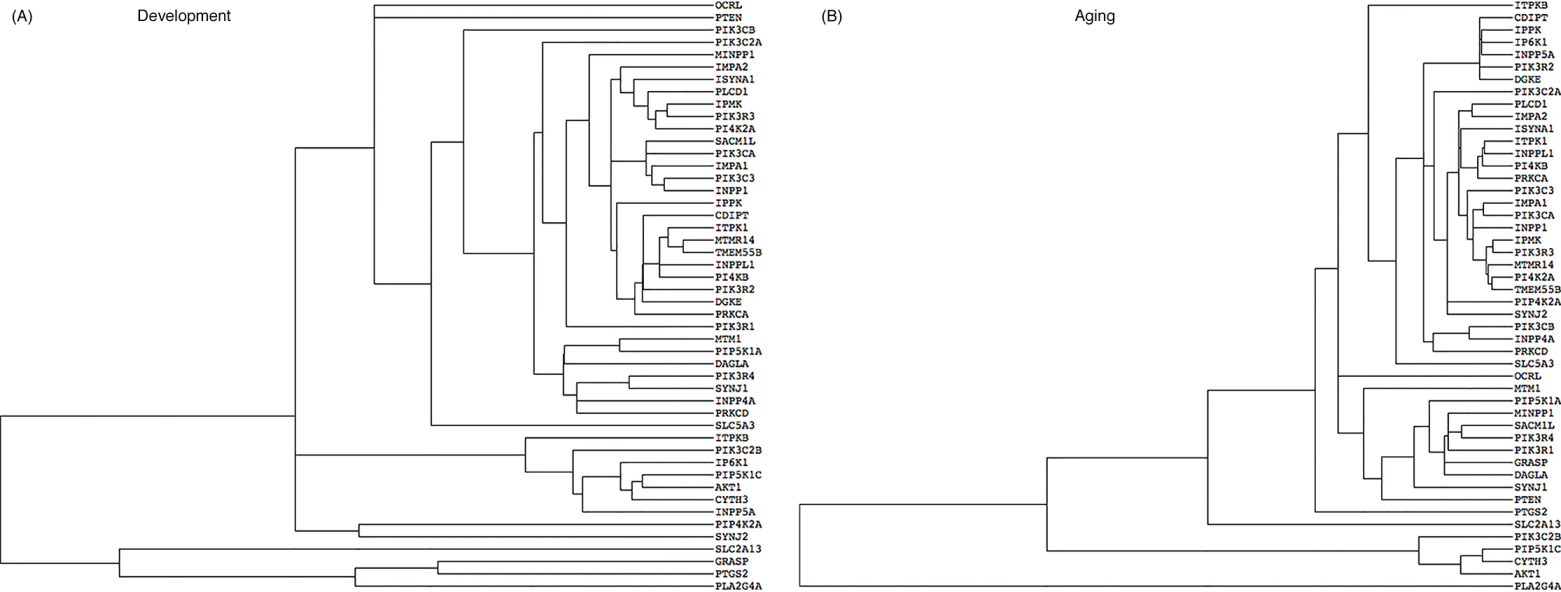
Hierarchical clustering of gene expression during Development (A) and Aging (B) intervals. Horizontal length indicates relative relatedness of age-dependent gene expression levels based on cluster calculations.

TreeView dendrograms can identify genes whose transcription is coordinated or clustered in a hierarchical cascade, indicating relatedness and common cellular processes [29, 30, 49]. For example, the Development dendrogram (Fig 3A) shows hierarchical interactions of *IMPA2* and *ISYNA1* (involved in *myo*-inositol synthesis); of *PLCD1*, *IPMK*, *PIK3R3*, and *PI4K2A*; and of *SACMIL*, *PIK3CA*, *IMPA1*, *PIK3C3*, and *INPP1*. *PLA2G4A*, *PTGS2 GRASP*, and *SLC2A13* are distant (more dissimilar expression patterns) from the other genes. The Aging dendrogram (Fig 3B) shows that *IMPA2* is closely tied to *PLCD1* and that *PLA2G4A* also is distant from the other genes.

**Fig 4:**
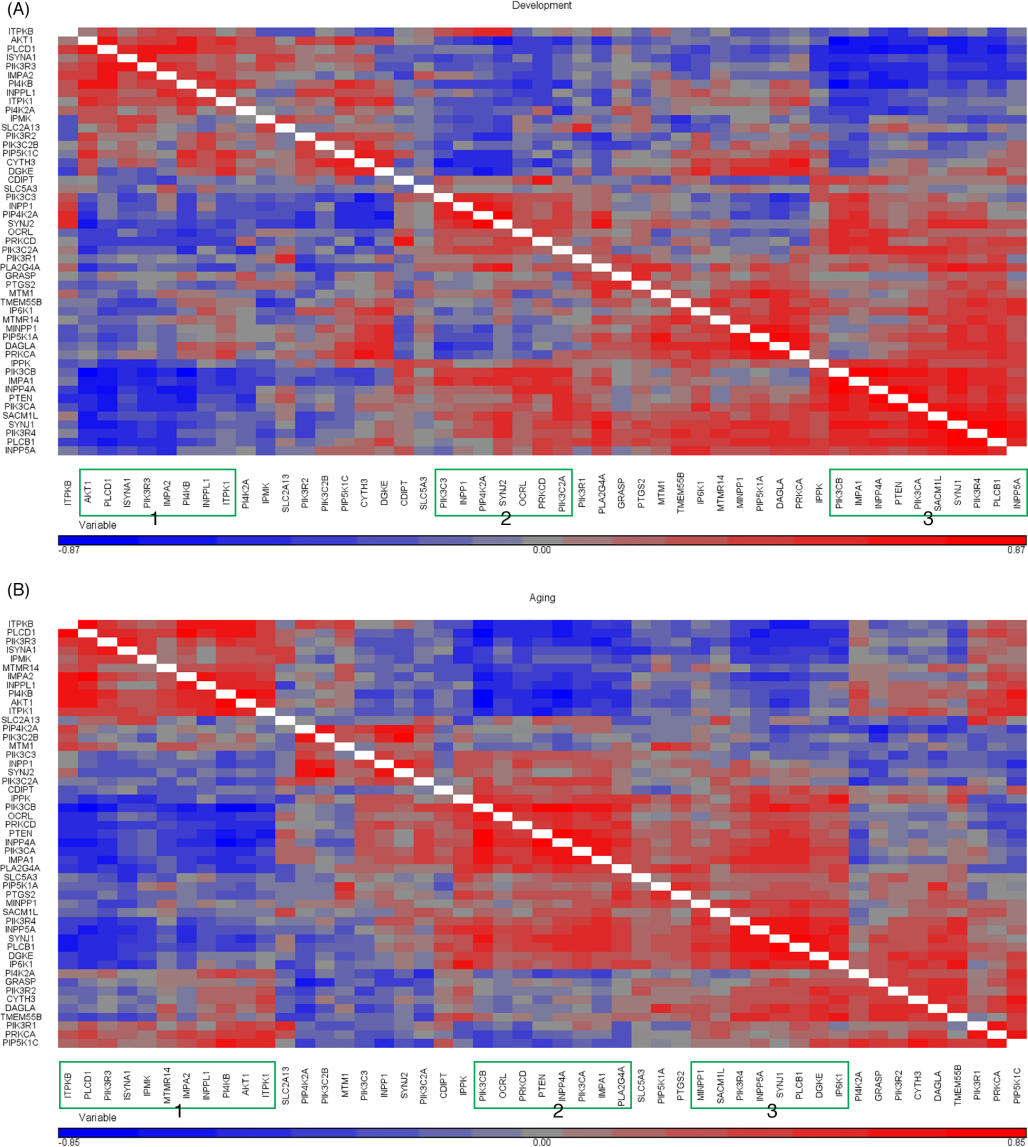
Heat maps for pairwise correlations of genes in Development (A) and Aging (B) intervals. Correlations or p-values are hierarchically clustered using the centroid linkage method.

Pearson’s correlation matrices (heat maps) of pairwise correlations among the 49 genes were created using unsupervised hierarchical clustering within the Development (Fig 4A) and Aging (Fig 4B) intervals. Hierarchical clustering row and column titles are not conserved between heat maps in the two intervals, as they represent the highest probability of correctly clustering genes based on Pearson’s r correlation in each interval. In the figures, genes that are positively intercorrelated within a cluster are highlighted in red, while those that are negatively intercorrelated are shown in blue.

Three distinct clusters of genes with highly positively intercorrelated expression levels are identified in both the Development and Aging intervals (green outlined boxes on x-axis). During Development (Fig 4A), the three groups are: *AKT1*, *PLCD1*, *ISYNA1*, *PIK3R3*, *IMPA2*, *PI4KB*, *INPPL1*, and *ITPK1* (Group 1); *PIK3C3*, *INPP1*, *PIP4K2A*, *SYNJ2*, *OCRL*, *PRKCD*, and *PIK3C2A* (Group 2); and *PIK3CB*, *IMPA1*, *INPP4A*, *PTEN*, *PIK3CA*, *SACM1L*, *SYNJ1*, *PIK3R4*, *PLCB1*, and *INPP5A* (Group 3).

S1A Fig presents correlation values corresponding to Fig 4A, and identifies correlations significant at p < 0.001 with green highlighting. It shows that genes within each of the three separate groups of Fig 4A are significantly intercorrelated. Genes in Group 1 are highly and negatively correlated with genes in Group 2. Genes in Group 1 and Group 3 also are highly and negatively correlated across groups, while genes in Group 2 and Group 3 are positively correlated across groups.

The heat map for Aging (Fig 4B) also identifies three distinct groups of intercorrelated genes, with many similarities (bolded) to the respective Development groups in Fig 4A: ***ITPKB***, ***PLCD1***, ***PIK3R3***, ***ISYNA*** *1*, *IPMK*, *MTMR14*, ***IMPA2***, ***INPPL1***, ***PI4KB***, and ***AKT1*** (Group 1); ***PIK3CB***, *OCRL*, *PRKCD*, ***PTEN***, *INPPLA*, ***PIK3CA***, ***IMPA1*,** and *PLA2G4A* (Group 2); and *MINPP1*, ***SACM1L***, ***PI3KR4***, ***INPP5A***, ***SYNJ1***, ***PLCB1*,** *DGKE*, and *IP6K1* (Group 3). The matrix of corresponding exact correlation values at p < 0.001 (S1B Fig) shows similar patterns to what is found during Development. Thus, genes within each of the three groups are significantly intercorrelated. Genes in Group 1 are negatively correlated with genes in Group 2, and genes in Group 3 are negatively correlated with *ITPKB*, *PLCD1*, *PIK3R3*, *ISYNA1*, and *IPMK* in Group 1. Genes in Groups 2 and 3 are positively correlated across groups.

## Discussion

Based on the literature, we constructed a phenotypic cascade containing known pathways of brain phosphoinositide metabolism and identified 49 genes whose protein products participate in this cascade (Fig 1). Among the 49 genes, we identified three groups in both Development and Aging and showed that the groups have similar intercorrelations and partially overlapping composition in the two intervals. In both intervals, *ITPKB*, *PLCD1*, *PIK3R3*, *ISYNA1*, *IMPA2*, *INPPL1*, *PI4KB*, and *AKT1* are Group 1, *PIK3CB*, *PTEN*, *PIK3CA*, and *IMPA1* are in Group 2, and *SACM1L*, *PI3KR4*, *INPP5A*, *SYNJ1*, and PLCB1 are in Group 3. Genes in Group 1 are negatively correlated with genes in Groups 2 and 3. Genes in Groups 2 and 3 are positively correlated across groups.

The similar gene groups and their interrelations in Development and Aging suggest that stable transcriptional networks underlie brain phosphoinositide metabolism throughout the entire lifespan, despite marked phenotypic differences between the two intervals [18–24]. Such stable networks likely arose through evolutionary constraints that maximized functional efficiency and minimized energy requirements of the metabolic pathways that were regulated [3, 50, 51]. A number of mechanisms may have contributed to their elaboration, such required coupling of reaction products and enzymes, enzyme colocalization at a cellular cite, organization of the nucleosome to bring genes and promoter regions together for co-transcription, and gene colocalization on common chromosomal bands [29, 32, 52].

We considered gene colocalization on the same chromosomal band in Table 3. Thus, expression levels correlate during Development and Aging for *PLA2G4A* and *PTGS2* colocalized on 1q25, consistent with evidence that arachidonic acid may have to be liberated from phospholipid by PLA_2_ before it can be oxidized to PGE_2_ by COX-2 [53, 54]. Levels for *PIK3R4* and *PIK3CB* on 3q22 and for *ITPK1* and *AKT1* on 14q32 also correlate significantly in the two age intervals. Levels for *PLCD1*, *SACMIL*, *IP6K1*, and for *PRKCD* on 3p21-23 correlate during Development and/or Aging, and levels for *DGKE and PRKCA* on 17q22 correlated during Development.

The dendrograms of Fig 4 identify certain gene expression hierarchies in the two intervals. For example, the Development dendrogram (Fig 4A) shows hierarchical relations among *IMPA2*, *ISYNA1*, *PLCD1*, *IPMK*, *PIK3R3*, and *PI43K*, which are distant from *PLA2G4A* and *PTGS2* within the arachidonic acid cascade [29]. *PLA2G4A* also is separated from the phosphoinositide hierarchy in the Aging dendrogram (Fig 3B).

Our separating Aging and Development in this study is consistent with our prior studies using BrainCloud [29, 30], and with phenotypic differences between the two intervals [18–24]. Of the 49 genes studied, 15 have a higher and 5 genes a lower mean expression level during Aging than Development (Table 1). Significant age correlations also are fewer during Aging than Development (Table 2), but lesser variation during Aging may have reduced our power to determine statistically significant correlations [55].

Nonlinear increases in *ITBKB*, *PIP4K2A*, *SYNJ2*, *PRKCD*, *GRASP*, *PLA2G4A* and *PTGS2* expression and decreases in *PIK3C2B*, *CYTH3*, and *DGKE* expression during Development likely correspond to the many nonlinear phenotypic changes that have ben described, suggesting a role for phosphoinositides in them. There is rapid neuronal loss in the first year of life, dendritic growth followed by pruning over a 15 year period, declining myelination, changing arachidonic and docosahexaenoic acid concentrations throughout the entire interval, and conversion from ketone body to glucose use for oxidative metabolism in the first year [18–24].

Some of our correlations are consistent with reported enzyme coupling in phosphoinositide enzyme pathways (Fig 1). For example, PTEN (phosphatidylinositol 3,4,5-trisphosphate 3-phosphatase) dephosphorylates PI(3,4,5)P_3_ to PI(4,5)P_2_ and negatively influences both AKT (*AKT1*) and PI(3,4,5)P_3_ signaling [56]. *PTEN* and *AKT1* are in separate groups that are correlated negatively in the Development and Aging heat maps (Fig 4A and 4B). Aberrant activation of neuronal PI3K/AKT/mTOR and PTEN signaling may be an early prelude of Alzheimer disease [57, 58].

The association between *PLCD1* and *IMPA2* in Group 1 in each age interval also is of interest because IMPA2, which dephosphorylates *myo*-inositol monophosphate within the “PI cycle”, has been proposed as the preferred target of lithium in the treatment bipolar disorder [59–61]. The PI cycle is initiated by PLC-hydrolysis of PI(4,5)P_2_, followed by formation Ins(1,4,5)P_3_, Ins(1,4)P_2_, Ins(4)P, *myo*-inositol, phosphatidylinositol (PI), PI(4)P and finally resynthesis of PI(4,5)P_2_ [43, 60].

During Development, the positive age correlations of *SYNJ1* and *SYNJ2* expression (Table 2), whose protein products modify clathrin-mediated synaptic endocytosis [2, 39], correspond to a reported increase in SYNJ immunoreactivity and dendritic spine density in brain [19, 62]. *SYNJ2* expression is 1.8 fold higher in Aging than Development, suggesting late stage synaptic changes [63]. Finally, *SYNJ1*, *SYNJ2*, and *INPP1* are in Group 3 with *PTEN* in both intervals. It is reasonable that genes whose proteins degrade PI(3,4,5)P_3_ should be positively correlated with *PTEN*, because if PTEN negatively regulates PI(3,4,5)P_3_ signaling it could do so by interacting with other enzymes that degrade PI(3,4,5)P_3_.

The 1.67 fold decrement in *SLC2A13* (HMIT) expression in Aging compared with Development does not correspond to a decreased brain *myo*-inositol concentration [64, 65]. However, the negative age correlation in *GRASP* expression (Table 2) during Aging suggests glutamatergic alterations, since GRASP links Group 1 metabotropic glutamate receptors to neuronal proteins [66]. The highly positive correlation of *ITPKB* expression during Aging differs from a report that *ITPKB* mRNA was not increased with age in postmortem brain [67].

Class IA PI3K dimers evolved from a single enzyme in unicellular eukaryotes [68]. They consist of a p110 catalytic and a p85 regulatory subunit, each of which has three isoforms, PIK3CA, PIK3CB, and PIK3CD, and PIK3R1, PIK3R2 and PIK3R3, respectively [68]. Expression of *PIK3R2* decreases while that of *PIK3R4* increases during Development (Table 2), demonstrating the principle of divergent expression after gene duplication [69]. The regulatory subunit *PIK3R3* is in Group 1 while the catalytic subunits *PIK3CA* and *PIK3CB* are in Group 3 in both the Development and Aging heat maps and the genes in the two groups are negatively correlated.

Although BrainCloud is a powerful tool for examining gene expression changes with age, it has limitations. It only contains data for the dorsolateral prefrontal cortex [26], but expression patterns differ between brain regions [70]. It also does not distinguish between cell types, which can also have distinct transcriptional trajectories [27, 28, 70]. However, BrainCloud does have a large number of samples, which increases its statistical power. The Allen Human Brain Atlas contains data only from 3 men, while the Loerch et al. study contains 28 brain samples [26–28].

In the future, it would be of interest to investigate mechanisms underlying coordinated transcription in relation to changing levels of the transcribed proteins. Methylation of gene promoters, histone acetylation and methylation state, transcription factors, miRNAs, DNA sequences of cis-elements (transcription factor binding sites), and feedback regulation by metabolites can influence expression [71–73]. Genes whose expression decreases with age appear to have higher promoter GC content than the other genes [71], suggesting differences in methylation state [74].

In summary, we hade described coordinated changes during Development and Aging in transcription of genes coding for multiple aspects of brain phosphoinositide metabolism, suggesting important roles for these genes. Three somewhat similar groups of genes with distinct expression intercorrelations were identified in each of the two intervals, and some pairwise correlations could be related to colocalization on the same chromosomal band. Nonlinear changes during Development likely participate in concurrent nonlinear phenotypic changes within this period. Mechanisms of coordinated transcription in normal as well as pathological human brain deserve to be explored further.

## Supporting Information

S1 Fig Matrices of correlations highlighted with significance between gene pairs corresponding to heat maps during Development (A) and Aging (B). Correlations highlighted in green are significant at p < 0.05 and p < 0.001 shown on separate tabs.
(XLSX)
